# Impact of different stages of intrauterine inflammation on outcome of preterm neonates: Gestational age-dependent and -independent effect

**DOI:** 10.1371/journal.pone.0211484

**Published:** 2019-02-08

**Authors:** Carlo Pietrasanta, Lorenza Pugni, Daniela Merlo, Barbara Acaia, Dario Consonni, Andrea Ronchi, Manuela Wally Ossola, Beatrice Ghirardi, Ilaria Bottino, Fulvia Milena Cribiù, Silvano Bosari, Fabio Mosca

**Affiliations:** 1 NICU Fondazione IRCCS Ca’ Granda Ospedale Maggiore Policlinico, Milan, Italy; 2 University of Milan, Department of Clinical Sciences and Community Health, Milan, Italy; 3 Pathology Unit, Fondazione IRCCS Ca' Granda Ospedale Maggiore Policlinico, Milan, Italy; 4 Gynecology and Obstetrics Unit, Fondazione IRCCS Ca' Granda Ospedale Maggiore Policlinico, Milan, Italy; 5 Epidemiology Unit, Fondazione IRCCS Ca' Granda Ospedale Maggiore Policlinico, Milan, Italy; 6 University of Milan, Department of Pathophysiology and Transplantation, Milan, Italy; University of Missouri Columbia, UNITED STATES

## Abstract

**Objective:**

To investigate the impact of different stages of intrauterine inflammation (IUI) on neonatal outcomes, before and after adjusting for gestational age (GA) and other perinatal confounders.

**Methods:**

This was an observational, prospective, single-center cohort study including all eligible neonates with GA < 35 weeks and/or birth weight ≤ 1500 g born at a 3^rd^ level Neonatal Intensive Care Unit between 2011 and 2014. Pathological patterns of placenta, membranes and cord were classified according to Redline’s criteria. Multivariable linear and logistic regression models were applied, either including or not GA among the covariates.

**Results:**

Of the 807 enrolled neonates, 134 (16.6%) had signs of IUI: among these, 54.5% showed just histological chorioamnionitis (HCA), 25.4% had HCA + funisitis (FUN) stage 1, and 20.1% had HCA + FUN stage 2–3. At univariate analysis, HCA increased the risk for retinopathy of prematurity (ROP) and bronchopulmonary dysplasia, while FUN (any stage) had a deleterious impact on all outcomes investigated. After adjustment for covariates not including GA, HCA was a risk factor only for ROP (OR = 2.8, CI: 1–7.8), while FUN (any stage) was still associated with increased ORs for all outcomes (*p* <0.01). Upon inclusion of GA in the regression model, the results differed remarkably. HCA was associated with lower risk for mechanical ventilation (OR = 0.3, CI: 0.1–0.7) and need for surfactant (OR = 0.5, CI: 0.2–0.9), while FUN (any stage) worsened clinical conditions at birth (*p* <0.05), increased the risk for early-onset sepsis (*p* <0.01), and increased the length of mechanical ventilation (FUN stage 2–3 only, RC = 6.5 days, CI: 2–11). No other outcome was affected.

**Conclusions:**

IUI, especially FUN, negatively impact most neonatal morbidities, but its effect is partially reverted adjusting for GA. Considered that GA is an intermediate variable interposed between prenatal causes of prematurity and outcomes, the appropriateness of adjusting for GA may be questionable.

## Introduction

Acute perinatal intrauterine inflammation (IUI) is one of the leading causes of preterm birth worldwide and its prevalence increases with decreasing gestational age (GA): up to 70% of preterm births at 23–25 weeks of GA are associated with IUI [1–3]. IUI may involve both maternal and fetal compartment, with signs of inflammation limited to the membranes chorion and amnion (maternal inflammation, referred to as histological chorioamnionitis, HCA) or also within the walls of umbilical cord vessels (fetal inflammation or funisitis, FUN) [4]. These histopathological patterns still represent the gold standard for the diagnosis of IUI and few different classification systems have been proposed over the past 30 years [5–8].

To diagnose IUI before or immediately after birth, earlier than histopathological examination, the use of several clinical and biochemical signs has been proposed [9–14]. Nonetheless, none of them nor any specific combination have been proved to provide acceptable sensitivity and specificity for the diagnosis of IUI up to now, while the new descriptive term of “intrauterine inflammation or infection or both”, abbreviated as “Triple I”, has been recently proposed to harmonize different clinical definitions and replace the term chorioamnionitis in the clinical context [15,16].

Although IUI is a well-known cause of preterm birth, the correlation between its different stages or grades and neonatal adverse outcomes is far from being clarified [17]. In the last decades, numerous studies have attempted to establish whether, and to what extent, IUI might negatively affect the short- and long-term outcome of preterm neonates. Several conditions peculiar of prematurity, such as respiratory distress syndrome (RDS), bronchopulmonary dysplasia (BPD) [18,19], neurological short- and long-term adverse outcomes [20–23], sepsis [24,25], retinopathy of prematurity (ROP) [26], and the patency of ductus arteriosus (PDA) [27] have been variably correlated to either a clinical or histological diagnosis of IUI, with discordant results. The inconsistency observed across studies can be attributed largely to the enrollment of different study populations, the use of different diagnostic criteria and methods, and to whether or not potential confounding factors were taken into account. Particularly, although most studies considered GA at birth among other confounding factors, few authors clearly explored the role of GA independently of other covariates.

Considering controversial results among existing studies, we decided to perform a study to investigate the importance of different stages of perinatal IUI, histologically diagnosed, on neonatal outcomes of preterm birth, before and after adjusting for antenatal and perinatal variables and with a specific focus on the adjustment for GA.

## Materials and methods

### Study population and design

This was an observational, prospective, single-center study conducted at the Neonatal Intensive Care Unit (NICU) of Fondazione IRCCS Ca’ Granda Ospedale Maggiore Policlinico of Milan, Italy, between November 2011 and December 2014. The study protocol was approved by the Ethics Committee of the Hospital (Comitato Etico Milano Area B—Fondazione IRCCS Ca’ Granda Ospedale Maggiore Policlinico Milano). Written informed consent was obtained from parents for the inclusion in the study, and all procedures were in accordance with the Helsinki Declaration of 1975, as revised in 2008.

All inborn neonates with a GA <35 weeks and/or a birth weight (BW) ≤1500 g admitted to the NICU were consecutively enrolled in the observational study. Exclusion criteria were being outborn, the presence of major congenital anomalies, lack of parental consent or a missing pathological examination of fetal adnexa.

Histopathological examination of placenta, chorioamniotic membranes and umbilical cord was performed by a single pathologist expert in perinatal pathology, aware of GA and BW but blinded to other clinical data. At least 7 samples were collected, included in paraffin, sectioned and stained with hematoxylin and eosin: 1 samples from the membranes, 3 from the umbilical cord, and 3 from the placental disc, plus additional samples from macroscopically abnormal areas. Pathological patterns of placenta, membranes and cord were described and classified according to Redline’s criteria [7].

Neonates were classified into four groups, based on the result of pathological examination of fetal adnexa: Group 0 (controls, no inflammation detected), Group 1 (HCA, presence of HCA of any stage or grade, without involvement of umbilical cord or chorionic plate vessels), Group 2 (HCA + FUN 1, presence of HCA plus FUN stage 1), and Group 3 (HCA + FUN 2–3, presence of HCA plus FUN stage 2–3).

### Data collection and definitions

Data from mothers and neonates were prospectively collected using an electronic database. The following maternal variables were recorded: age at delivery, ethnicity, premature (at least 1 hour before the onset of contractions)–rupture of the membranes (PROM), PROM >24 hours, clinical chorioamnionitis, antibiotic therapy in labor, prenatal steroids, pre-eclampsia. A preterm birth was defined as “indicated” in case of induced vaginal delivery or cesarean section in the absence of preterm labor. Clinical chorioamnionitis was defined as the presence of at least two of the following criteria during labor, in absence of other known cause: maternal fever >38°C, maternal leukocytosis >15,000 leukocytes/mm^3^, maternal C-reactive protein (CRP) >10 mg/dL, foul-smelling or purulent amniotic fluid, persistent maternal heart rate >100 bpm or fetal heart rate >160 bpm.

Moreover, we recorded the following neonatal data: GA (dated by first trimester ultrasound crown-rump length measurement, when available, or calculated from the first day of last menstrual period), BW, sex, mode of delivery, twins, small for gestational age (SGA) defined as BW <10^th^ percentile according to Fenton [28], need for resuscitation (at least ventilation with mask), supplemental oxygen, intubation and surfactant at birth, Apgar score at 1 and 5 minutes of life, presence of RDS, total doses of surfactant, duration of invasive and non-invasive ventilation (including heated humidified high flow nasal cannulae), occurrence of culture-proven early-onset (within the first 72 hours of life) sepsis (EOS), late-onset (after the first 72 hours of life) sepsis (LOS), PDA, intraventricular hemorrhage (IVH), ROP, BPD, and death during hospitalization.

RDS was defined as PaO2 <50 mmHg in room air, central cyanosis in room air, a requirement for supplemental oxygen to maintain PaO2 >50 mmHg, or a requirement for supplemental oxygen to maintain a pulse oximeter saturation over 85% within the first 24 hours of life, and a chest radiograph consistent with RDS (reticulogranular appearance to lung fields with or without low lung volumes and air bronchograms) within the first 24 hours of life [29]. BPD was defined according to Jobe’s criteria [30]. IVH was defined according to Volpe’s classification by head ultrasound scan [31], and ROP was defined according to the international classification for retinopathy of prematurity [32].

### Statistical analysis

We calculated regression coefficients (RCs), odds ratios (ORs), 95% confidence intervals (CIs), and two-sided *p*-values for each of the three groups with IUI in comparison with the control group using univariate and multivariable linear (for quantitative variables) or logistic (for dichotomous variables) regression models. Variables included in regression models were selected a priori. To investigate the role of GA independently of the other covariates (maternal age, ethnicity, antenatal steroids, sex, SGA neonate, and indicated preterm birth), models including GA at delivery were compared with those without GA at delivery. Standard errors of RCs and log(ORs) were adjusted to take into account the outcome correlations within twin births. Statistical analysis was performed using Stata 15 (StataCorp. 2017, College Station, TX).

## Results

During the study period a total of 1024 neonates with GA <35 weeks and/or BW ≤1500 g were born at the study site. Of these, 217 (21.2%) were excluded because of unavailable placental examination or major congenital malformation. A total of 807 babies were enrolled, with mean GA 31.9 ± 2.7 weeks and mean BW 1656 ± 535 g. Females were 47.1%. The full dataset is available in supporting information (S1 File).

Of the 807 enrolled neonates, 134 (16.6%) had signs of fetal adnexa inflammation: among these, 73 (54.5%) showed just HCA, 34 (25.4%) had signs of HCA + FUN 1, and 27 (20.1%) had signs of HCA + FUN 2–3. The incidence of both HCA alone and FUN decreased progressively with increasing GA, dropping from 12.5% at 22–24 weeks of gestation to 2.4% at 33–34 weeks (HCA) and from 45.8% at 23–24 weeks to 8% at 33–34 weeks (FUN, any stage), respectively.

The neonates born to mothers with any IUI had lower GA and lower BW compared to the control group, and a stepwise reduction in both GA and BW was correlated with increasing stages of IUI, from chorioamnionitis to the highest stages of fetal response (all *p* <0.05 compared to the control group, Table 1). A progressive decrease in the incidence of caesarean section and pre-eclampsia was recorded from neonates born without HCA (90.1% and 14.1%, respectively) to neonates with FUN stage 2–3 (59.3% and 0, respectively). A similar, stepwise decrement was found for indicated preterm birth (from 57.8% in the absence of IUI to 3.7% among neonates with FUN stage 2–3, p <0.01), while the incidence of PROM, clinical chorioamnionitis and administration of peripartum antibiotics increased together with the severity of IUI. Particularly, clinical signs of maternal chorioamnionitis were recorded in 81.5% of neonates with HCA + FUN stage 2–3, 47.1% of neonates with FUN stage 1, and 15.1% of neonates with HCA alone (all *p* <0.01 compared to the control group) (Table 1).

**Table 1:** Maternal and perinatal characteristics of enrolled neonates.

	*No IUI* *Group 0* *n = 673*	*HCA* *Group 1* *n = 73*	*HCA+Fun 1 Group 2* *n = 34*	*HCA+Fun 2–3 Group 3* *n = 27*	*P value* *(1 vs 0)*	*P value* *(2 vs 0)*	*P value* *(3 vs 0)*
**Maternal age** **(mean ± SD)**	35.1 ± 6.1	36.3 ± 5.8	34 ± 5.7	33.4 ± 7.3	0.93	0.59	0.34
**Caucasian ethnicity (%)**	578/673 (86)	63/73 (86.3)	27/24 (79.4)	14/27 (51.9)	0.93	0.31	<0.01
**PROM (%)**	208/673 (30.9)	38/73 (52)	21/34 (61.7)	26/27 (96.3)	<0.01	<0.01	<0.01
**PROM >24 h (%)**	75/208 (36.1)	20/38 (52.6)	10/21 (47.6)	18/26 (69.2)	0.07	0.32	<0.01
**Indicated preterm birth (%)**	389/673 (57.8)	27/73 (37)	2/34 (5.9)	1/27 (3.7)	<0.01	<0.01	<0.01
**Clinical choriamnionitis (%)**	25/671 (3.7)	11/73 (15.1)	16/34 (47.1)	22/27 (81.5)	<0.01	<0.01	<0.01
**Maternal antibiotics (%)**	192/673 (28.5)	39/73 (53.4)	20/34 (58.8)	24/27 (88.9)	<0.01	<0.01	<0.01
**Prenatal steroids (%)**	524/671 (78.1)	63/73 (86.3)	25/34 (73.5)	26/27 (96.3)	0.14	0.57	0.05
**Pre-eclampsia (%)**	95/673 (14.1)	5/73 (6.8)	1/34 (2.9)	0/27	0.09	0.1	n.a.
**Caesarean section (%)**	605/673 (90.1)	63/73 (86.3)	24/34 (70.6)	16/27 (59.3)	0.35	<0.01	<0.01
**Male (%)**	351/673 (52.1)	46/73 (63)	17/34 (50)	13/27 (48.1)	0.09	0.82	0.68
**SGA (%)**	115/673 (17.1)	17/73 (23.3)	0/34	0/27	0.18	n.a.	n.a.
**Twin (%)**	361/673 (53.6)	30/73 (41.1)	14/34 (41.2)	4/27 (14.8)	0.08	0.21	<0.01
**Gestational age** **(mean ± SD)**	32.3 ± 2.5	31.4 ± 3.1	28.9 ± 3.4	28.3 ± 3.8	<0.01	<0.01	<0.01
**Birth weight** **(mean ± SD)**	1704 ± 518	1492 ± 501	1385 ± 640	1235 ± 562	<0.01	<0.02	<0.01

n.a.: not applicable

FUN, both at stage 1 and stage 2–3, was associated with significantly lower Apgar scores at 1 and 5 minutes of life, and an increased need for resuscitation and oxygen in the delivery room compared to the control group (all *p* <0.01, Table 2).

**Table 2:** Incidence of neonatal outcomes and unadjusted linear (quantitative outcomes) or logistic (dichotomous outcomes) regression analysis.

	*No IUI* *Group 0* *n = 673*	*HCA* *Group 1* *n = 73*	*HCA+Fun 1* *Group 2* *n = 34*	*HCA+Fun 2–3* *Group 3* *n = 27*	*RC/ORs and CI* *(1 vs 0)*	*RC/ORs and CI* *(2 vs 0)*	*RC/ORs and CI* *(3 vs 0)*
**Apgar at 1 min** **(median, range)**	8, 0–10	8, 0–9	5,5, 1–9	5, 1–9	-0.3 -0.8; 0.1	-1.5 -2.1; -0.8	-2.5 -3.2; -1.7
**Apgar at 5 min** **(median, range)**	9, 2–10	9, 3–10	8, 5–10	8, 3–10	-0.2 -0.6; 0.1	-0.8 -1.2; -0.3	-1.4 -1.8; -0.9
**Resuscitation at birth (%)**	320/673 (47.5)	40/73 (54.8)	31/34 (91.2)	24/27 (88.9)	1.3 0.8; 2.2	11.4 3.4; 38.3	8.8 2.6; 29.6
**Oxygen in delivery room (%)**	175/672 (26)	25/73 (34.2)	24/34 (70.5)	19/27 (70.4)	1.5 0.8; 2.6	6.8 3.1; 14.5	6.7 2.9; 15.7
**Mechanical ventilation (%)**	128/673 (19)	13/73 (17.8)	19/34 (55.8)	14/27 (51.8)	0.9 0.4; 1.8	5.4 2.6; 11.1	4.6 2.1; 10.1
**Mechanical ventilation (days)**	2.1	3.9	8.5	15.6	1 -1.8; 3.8	5.4 1.4; 9.5	12.2 7.6; 16.7
**Non-invasive ventilation (%)**	364/673 (54.1)	39/73 (53.4)	26/34 (76.5)	20/27 (74.1)	0.9 0.6; 1.6	2.8 1.2; 6.1	2.4 1; 5.8
**Non-invasive ventilation (days)**	10.8	16.3	30	26.7	2.6 -3.9; 9	11.9 2.6; 21.3	13.6 2.9; 24.2
**Surfactant use (overall)**	204/673 (30.3)	21/73 (28.8)	19/34 (55.9)	17/27 (62.9)	0.9 0.5; 1.7	2.9 1.5; 5.7	3.9 1.7; 8.7
**Surfactant use (>1 dose)**	67/673 (9.9)	9/73 (12.3)	10/34 (29.4)	11/27 (40.7)	1.2 0.5; 2.9	4.6 2; 10.7	7.7 3.1; 18.9
**RDS** **(%)**	341/673 (50.7)	41/73 (56.2)	30/34 (88.2)	23/27 (85.2)	1.2 0.7; 2.1	7.3 2.5; 21.3	5.6 1.9; 16.4
**EOS** **(%)**	3/673 (0.45)	1/73 (1.37)	4/34 (11.8)	2/27 (7.41)	3.1 0.3; 30.3	29.8 6.3; 100	17.8 2.8; 100
**LOS** (%)*	43/672 (6.4)	8/72 (11.1)	6/33 (18.2)	8/27 (29.6)	1.8 0.8; 4.4	3.3 1.3; 8.2	6.2 2.5; 15
**PDA** **(%)**	72/673 (10.7)	10/73 (13.7)	11/34 (32.4)	12/27 (44.4)	1.3 0.6; 2.7	4 1.9; 8.4	6.7 2.9; 15
**IVH** **(%)**	40/673 (5.9)	7/73 (9.59)	9/34 (26.5)	9/27 (33.3)	1.7 0.6; 4.2	5.7 2.3; 13.7	7.9 3.3; 18.9
**ROP** (%)*	21/661 (3.2)	7/70 (10)	6/30 (20)	7/25 (28)	3.4 1.2; 9.1	7.6 2.5; 23.2	11.8 4.4; 31.8
**BPD** (%)*	60/656 (9.2)	13/70 (18.6)	9/28 (32.1)	10/23 (43.5)	2.3 1.1; 4.7	4.7 1.9; 11.8	7.6 3.2; 18.4
**Death before discharge (%)**	19/673 (2.8)	5/73 (6.9)	6/34 (17.6)	5/27 (18.5)	2.5 0.9; 6.9	7.4 2.7; 19.9	7.8 2.6; 23.1

*percentage is calculated on survived neonates

Both early and advanced stages of FUN had a deleterious impact on neonatal outcomes, increasing the ORs for invasive and non-invasive ventilation, the need for surfactant, and the incidence of RDS, EOS, LOS, PDA, IVH, ROP, BPD, and death (all *p* <0.05, Table 2). Maternal HCA alone had a much more limited effect on neonatal outcomes, with an increased OR for ROP (OR = 3.4, 95% CI = 1.2–9.1, *p* = 0.02) and BPD (OR = 2.3, 95% CI = 1.1–4.7, *p* = 0.03).

Upon multivariable analysis without GA as a covariate (Table 3), HCA alone was not significantly associated with any neonatal adverse outcome, except for an increased risk for ROP (OR = 2.8, 95% CI = 1–7.8, *p* = 0.04). Conversely, neonates with histological signs of FUN, independently of the stage, showed worse adaptation at birth, with lower Apgar scores, increased need for resuscitation/oxygen in the delivery room, and increased ORs for mechanical ventilation, surfactant use, RDS, EOS, LOS, PDA, IVH, ROP, BPD, and death before discharge (all *p* <0.01). Only the need for non-invasive ventilation was less affected, with not significant ORs in neonates with FUN stage 2–3.

**Table 3:** Multivariable regression analysis of neonatal outcomes after adjustment for perinatal covariates^a^, excluding gestational age.

	*Adjusted RC/ORs* *(1 vs 0)*	*95% CI* *P value*	*Adjusted RC/ORs* *(2 vs 0)*	*95% CI* *P value*	*Adjusted RC/ORs* *(3 vs 0)*	*95% CI* *P value*
Apgar at 1 min (points)*	-0.2	(-0.7; 0.2) 0.3	-1.5	(-2.2; -0.8) < 0.01	-2.2	(-2.9; -1.4) < 0.01
Apgar at 5 min (points)*	-0.2	(-0.5; 0.1) 0.2	-0.7	(-1.1; -0.3) <0.01	-1.2	(-1.7; -0.7) <0.01
**Resuscitation at birth**	1.2	(0.7; 2) 0.6	12.5	(3.7; 41.5) <0.01	6.2	(1.7; 22.2) <0.01
**Oxygen in delivery room**	1.4	(0.7; 2.6) 0.3	7.2	(3.3; 15.9) <0.01	5	(2; 12.5) <0.01
**Mechanical ventilation**	0.8	(0.4; 1.5) 0.5	5.4	(2.5; 11.8) <0.01	3.8	(1.6; 9.1) <0.01
Mechanical ventilation (days)*	0.6	(-2.2; 3.5) 0.7	5.4	(1.2; 9.5) 0.01	11.5	(6.7; 16.2) <0.01
**Non-invasive ventilation**	0.9	(0.5; 1.6) 0.75	2.9	(1.3; 6.7) 0.01	1.8	(0.7; 4.8) 0.2
**Surfactant use (overall)**	0.9	(0.5; 1.6) 0.7	3	(1.5; 5.9) <0.01	3.5	(1.5; 8.3) <0.01
**RDS**	1.2	(0.6; 2) 0.61	7.8	(2.6; 23) <0.01	4.3	(1.4; 13.5) 0.01
**EOS**	2.5	(0.3; 23.4) 0.46	89.9	(8.5; 100) <0.01	50	(8.7; 100) <0.01
**LOS**	1.6	(0.6; 4.1) 0.32	4.0	(1.5; 10.9) <0.01	5.9	(2; 17.9) <0.01
**PDA**	1.2	(0.6; 2.5) 0.65	4. 1	(1.8; 9.1) <0.01	6	(2.4; 14.7) <0.01
**IVH**	1.3	(0.6; 3.5) 0.46	4.3	(1.6; 11.4) <0.01	4.3	(1.7; 11.4) <0.01
**ROP**	2.8	(1.0; 7.8) 0.04	7.6	(2.4; 24) <0.01	8.8	(2.8; 27.7) <0.01
**BPD**	1.8	(0.8; 3.8) 0.14	4.7	(1.8: 12.8) <0.01	6	(2.3; 15.8) <0.01
**Death before discharge**	2.0	(0.7: 5.8) 0.17	7.3	(2.3: 23.7) <0.01	6.3	(1.9: 21) <0.01

^a^maternal age, ethnicity, sex, small for gestational age, antenatal steroids, indicated preterm birth

*regression coefficient (RC) has the same unit of measure of the variable

After the inclusion of GA in the regression model, the results differed remarkably (Table 4).

**Table 4:** Multivariable regression analysis of neonatal outcomes after adjustment for perinatal covariates^a^, including gestational age.

	*Adjusted RC/ORs* *(1 vs 0)*	*95% CI* *P value*	*Adjusted RC/ORs* *(2 vs 0)*	*95% CI* *P value*	*Adjusted RC/ORs* *(3 vs 0)*	*95% CI* *P value*
Apgar at 1 min (points)*	0	(-0.4; 0.4) 0.99	-0.6	(-1.2; -0.1) 0.05	-1	(-1.7; -0.4) <0.01
Apgar at 5 min (points)*	-0.1	(-0.3; 0.2) 0.6	-0.1	(-0.5; 0.2) 0.5	-0.5	(-0.9; -0.1) 0.03
**Resuscitation at birth**	0.8	(0.4; 1.6) 0.6	6.1	(1.4; 25.3) 0.01	2.5	(0.7; 8.5) 0.1
**Oxygen in** **delivery room**	1	(0.5; 2.1) 0.88	3.2	(1; 9.3) 0.03	2.2	(0.8; 5.6) 0.11
**Mechanical ventilation**	0.3	(0.1; 0.7) <0.01	1.9	(0.6; 6.2) 0.3	0.9	(0.2; 4.5) 0.94
Mechanical ventilation (days)*	-0.4	(-3.1; 2.4) 0.8	0.9	(-2.3; 4.5) 0.6	6.5	(2; 11) <0.01
**Non-invasive ventilation**	0.7	(0.4; 1.2) 0.2	1.1	(0.3; 3.9) 0.93	0.6	(0.2; 2.1) 0.46
**Surfactant use (overall)**	0.5	(0.2; 0.9) 0.05	0.6	(0.2;2.3) 0.59	1.2	(0.4; 3.5) 0.71
**RDS**	0.7	(0.4; 1.4) 0.4	2.8	(0.6; 12.5) 0.17	1.4	(0.4; 4.9) 0.59
**EOS**	2.4	(0.3; 18.7) 0.4	85	(9.4; 100) <0.01	37.6	(6.7; 100) <0.01
**LOS**	1.2	(0.3; 3.8) 0.8	0.9	(0.3; 2.7) 0.86	1.2	(0.2; 6.1) 0.83
**PDA**	0.6	(0.2; 1.6) 0.3	0.9	(0.3; 2.4) 0.83	1.8	(0.4; 8.3) 0.44
**IVH**	0.8	(0.3; 2.1) 0.65	1	(0.3; 3.6) 0.9	0.9	(0.2; 3.5) 0.9
**ROP**	3.3	(1; 10.2) 0.04	2	(0.4; 12.6) 0.4	1.7	(0.3; 11) 0.59
**BPD**	1.5	(0.6; 3.6) 0.42	1.2	(0.3; 4.5) 0.79	2.2	(0.2; 27.1) 0.57
**Death before discharge**	1.3	(0.4: 4.8) 0.65	2	(0.6: 7.1) 0.3	1.3	(0.4: 4.7) 0.4

^a^GA, maternal age, ethnicity, sex, small for gestational age, antenatal steroids, indicated preterm birth

*regression coefficient (RC) has the same unit of measure of the variable

HCA alone was protective against mechanical ventilation (OR = 0.3, 95% CI = 0.1–0.7, *p* <0.01) and against the need for exogenous surfactant (OR = 0.5, 95% CI = 0.2–0.9, *p* = 0.05); it was still associated with an increased risk for ROP (OR = 3.3, 95% CI = 1–10.2, *p* = 0.04). The effects of FUN were also greatly tempered, being limited to significantly reduced Apgar score at 1 minute of life (RC = -0.6, 95% CI = -1.2 to -0.1), increased need for resuscitation (OR = 6.1, 95% CI = 1.4 to 25.3) and oxygen in the delivery room (OR = 3.2, 95% CI = 1 to 9.3), and increased incidence of EOS (OR = 85, 95% CI = 9.4 to 100) for FUN stage 1; reduced Apgar scores (RC = -1, 95% CI = -1.7 to -0.4 for Apgar at 1 min; RC = -0.5, 95% CI = -0.9 to -0.1 for Apgar at 5 min), increased duration of mechanical ventilation (RC = 6.5, 95% CI = 2 to 11) and increased incidence of EOS (OR = 37.6, 95% CI = 6.7 to 100) for FUN stage 2–3. The correlation between any stage of FUN and all other clinically relevant neonatal outcomes was completely abolished after inclusion of GA in the regression model.

## Discussion

In the present study, we evaluated the impact of different stages of perinatal IUI on short-term outcomes of neonates less than 35 weeks’ gestation exposed to no IUI, HCA alone, and HCA plus FUN, before and after adjusting for antenatal and perinatal confounders and with a specific focus on the adjustment for GA.

We found an overall prevalence of histological IUI of 16.6%, slightly lower than that reported by other authors [33–36]. Some characteristics of the population studied, such as the wide GA range, the prevalent Caucasian ethnicity of the mothers, and the considerable proportion (51.9% overall) of cases of medically-induced preterm birth due to obstetric indications, e.g. multiple parity or intrauterine growth restriction, may at least partially explain this data [2]. FUN was associated with HCA in 45.5% of the placentas exhibiting signs of IUI, and it was always detected in association with maternal inflammation, confirming that intrauterine inflammatory process usually constitutes a “continuum” from mother to fetus. In line with the reports from other authors [33–37], both the incidence of HCA and FUN increased progressively with decreasing GA. Conversely, the incidence of indicated preterm birth was significantly lower in any IUI group compared with the control group, with a stepwise reduction according to the increase of IUI stage.

At univariate analysis, HCA increased the risk for ROP and BPD, while FUN (any stage) had a deleterious impact on all outcomes investigated. Then we performed two multivariable analyses with different regression models, including or not GA in the model. The purpose of this procedure was to highlight the role of GA adjustment in modifying correlations between risk factors, namely perinatal IUI, and neonatal outcomes, and to speculate on the appropriateness of adjusting for GA itself. The other covariates (maternal age, ethnicity, antenatal steroids, sex, SGA neonate, and indicated preterm birth), were selected a priori as they may affect, either positively or negatively, neonatal outcome. Without GA as a covariate, but still adjusting for the other confounders, results were not greatly affected compared to unadjusted analysis, especially for what concerns the effect of FUN. In fact, HCA alone was confirmed as a risk factor for ROP, but not anymore for BPD, while neonates born with both FUN stage 1 and stage 2–3 still had increased ORs for almost all the evaluated outcomes.

After inclusion of GA in the regression model, results differed dramatically. HCA alone was protective against the need for mechanical ventilation and surfactant, but it was confirmed as a risk factor for ROP. The reduced need for mechanical ventilation and surfactant, in the absence of a reduced OR for RDS, may support the hypothesis that a mild, just maternal perinatal inflammation would be protective against postnatal inflammatory stimuli inducing lung damage and subsequent need for ventilation, as highlighted by some authors [38,39]. Alternatively, considering that the risk of non-invasive ventilation was not affected by HCA, it could be hypothesized that IUI, accelerating lung development as demonstrated in several studies on animal models [40,41], causes less severe RDS requiring only non-invasive ventilation. As for the increased incidence of ROP, unexpectedly not confirmed in neonates with FUN, it has been suggested that perinatal inflammation may be involved in the pathogenesis of ROP [42]. However, a recent meta-analysis by Mitra and coll. [26] concluded that chorioamnionitis was significantly associated with ROP (any stage) as well as with severe ROP (stage ≥ 3) upon unadjusted analyses, but such association disappeared on subanalysis of the studies adjusting for GA. Certainly, the association between IUI and ROP is difficult to demonstrate due to many possible confounding factors, such as extreme prematurity and oxygen therapy [43].

Considering GA as a covariate in the logistic regression model, the relationship between FUN and neonatal outcome also changed considerably. FUN remained an independent risk factor for lower Apgar scores, an increased need for resuscitation at birth and oxygen in the delivery room (FUN stage 1), an increased number of days of mechanical ventilation (FUN stage 2–3), and for EOS (FUN any stage). In 2005, Lau and coll. [35], in a study conducted in a large cohort of neonates with a mean GA of 32–33 weeks admitted to the NICU, reported after adjusting for confounding factors an increased mortality and morbidity, including RDS, BPD, EOS, LOS, necrotizing enterocolitis (NEC), PDA, and IVH when chorioamnionitis was associated with a fetal inflammatory response. Conversely, most of the studies conducted in more recent years [36,37], including this one, failed to confirm or only partially confirmed these findings. In a retrospective study by Lee and coll. [37], conducted to evaluate in a relatively large group of neonates less than 34 weeks’ gestation if there was a stepwise increase in neonatal morbidities according to the stage or grade of acute HCA and FUN, the incremental trends of each neonatal outcome were found to be not significant after adjusting for confounding variables including GA at birth. In a subsequent study [36] on the relationship between all stages and grades of HCA and FUN and neonatal mortality and morbidity, only a high grade of fetal inflammation was significantly associated with BPD and NEC after adjusting for GA. Certainly, the increasingly widespread use of antenatal steroid may explain why some recent studies have failed to demonstrate an association between IUI and neonatal adverse outcomes. A meta-analysis published in 2011 [44] highlighted an association between antenatal corticosteroids administration and reduced incidence of RDS, IVH, and PDA, especially in the absence of maternal clinical chorioamnionitis. As a matter of fact, in the study by Lau and coll. prenatal steroids were administered in a percentage ranging from 40 to 60%, while in the most recent studies, including ours, they were administered in more than 80% of neonates.

As expected, we found a strong association between any stage of FUN and EOS even after including GA in the regression model. Contrary with what is seen for RDS, IVH, PDA and, albeit to a lesser extent, for BPD, antenatal steroids do not seem to have a protective effect on EOS [44]. Many studies have linked HCA, FUN, and even more clinical chorioamnionitis with EOS [21,35,45,46], although other studies have not confirmed this association [36,37], probably because maternal antibiotic therapy can affect culture results or the causative pathogen may be a mycoplasma, not identifiable with traditional culture methods.

Furthermore, in our study the presence of FUN stage 1 was strongly associated with an increased need for resuscitation and oxygen in the delivery room, independently of GA. This finding, not confirmed for FUN stage 2–3 possibly because of the small sample size, is not emphasized by many authors [35], and suggests that when the umbilical cord vessels are involved in inflammation, the preterm baby is very ill at birth and this can seriously worsen its outcome.

Lastly, the increased length of mechanical ventilation we found in the neonates with FUN stage 2–3, in the absence of higher odds for RDS and BPD, is not easy to interpret. We can hypothesize that the more severe clinical conditions at birth in neonates with FUN may cause a more severe acute lung disease, requiring prolonged mechanical ventilation before switching to a non-invasive ventilation. Moreover, the role of IUI in the development of neonatal pulmonary disease is far from being clarified. Despite evidence from animal models suggest a reduced incidence of RDS and an increased incidence of BPD in neonates exposed to IUI [47–49], the results of the numerous clinical studies on this topic are often contradictory. In particular, according to some authors, the presence of FUN in association with HCA appears to have a protective effect on the development of RDS [50–52] or BPD [1,53], findings not confirmed by other studies [35,36,54–56]. Regarding the development of BPD, the various insults (mechanical ventilation, oxygen, infection) that can damage the lung after birth must be considered. Some authors agree that the fetal lung, exposed to a prolonged pro-inflammatory stimulus in uterus, responds to postnatal inflammatory stimuli in a more pathologic way compared to a naïve lung [57]. On the other hand, studies on animal models have shown that a first exposure to an inflammatory stimulus can increase or decrease the response to a second injurious stimulus exposure depending on the time interval between exposures themselves [58,59].

The dichotomy between results obtained before and after adjustment for GA reflects the debate currently ongoing on the so called “independent role” of perinatal inflammation in shaping subsequent neonatal health [60]. As reported by Wilcox and coll. [61], GA is a consequence of any underlying cause of preterm birth, like IUI, and should be more properly considered as an “intermediate” variable rather than an “independent” variable. The adjustment for intermediate variables may in turn be misleading and favor a biased effect of other uncontrolled variables, rarely missing, that collide on the outcome. This bias can be so impactful that the relationship between IUI and neonatal adverse outcome may be even reversed after adjustment for GA. This issue has been also explored by other authors, like Dessardo and coll. [62], who tested a statistical approach incorporating path analysis to evaluate direct and indirect causality between exposure to HCA or FUN and the development of BPD. From this perspective, the inclusion of GA as a covariate, although recalling a common practice, may be strongly misleading.

Furthermore, the purpose of adjusting for GA is ideally to compare similar neonates with and without IUI, considering those without IUI as the control group. This assumption raises another subtle issue, because the components of this control group, when preterm neonates are involved, are rarely “control” subjects: rather they hide other pathological causes of preterm birth, first of all intrauterine growth restriction [2]. Thus, the effect of IUI on any neonatal outcome is not referred to healthy neonates, but to neonates born because of other causes of prematurity. The issue has been recently explored by Torchin and coll. [56] and Gagliardi and coll. [63], who demonstrated in large cohorts of preterm neonates that the apparent protective effect of IUI against the risk of developing BPD was due to the high rate, in the non-IUI group, of neonates affected by fetal growth restriction, a known predisposing condition for BPD according to the “vascular hypothesis”. Thus, the lack of a healthy control group in most studies may alter the real impact of IUI on neonatal outcomes. Interestingly, this issue seems to be neglected also in experimental models of IUI, where the effect of IUI itself is frequently investigated against a healthy, untreated control group, instead of a more appropriate group of animals with experimentally-induced fetal growth restriction.

The strengths of our study are rigorous definitions and staging of IUI, and prospective, single-center collection of data that reduces the variability sometimes affecting multicenter studies. We recognize some weaknesses of our work, such as the relatively small sample size, the wide GA range, and the relatively low incidence of IUI in our population.

In conclusion, our study shows that the influence of IUI on neonatal outcomes, without including GA among the covariates in multivariable regression analyses, is limited to an increased incidence of ROP, while FUN has a strong negative impact on most neonatal morbidities. With the inclusion of GA in regression models, results differ radically, with HCA reducing the need for mechanical ventilation and surfactant and FUN being a clear risk factor only for EOS and for worse clinical conditions in the delivery room. Considered that GA is an intermediate variable interposed between prenatal conditions favoring preterm delivery and neonatal outcomes, it is at least questionable whether the adjustment for GA, a common and accepted practice when investigating the correlation between IUI and neonatal outcomes, is methodologically correct. In order to better understand the independent role of IUI in shaping neonatal outcomes, we believe that further clinical studies and experimental models should not focus on GA adjustment or on the comparison between neonates affected or not by IUI but, rather, on a direct comparison between neonates affected by IUI and homogeneous groups of preterm neonates carrying other antecedents of preterm birth, such as placental vascular disorders, in order to have a more realistic picture of the different causes of prematurity and their really “independent” consequences.

## Supporting information

S1 File Spreadsheet containing all data included in the manuscript. Enlisted patients are completely anonymized.
(XLS)
